# Supplementary material for: The disturbance leg-lift response (DLR): an undescribed behavior in bumble bees
Source: PeerJ. 2021 Mar 25;9:e10997. doi: 10.7717/peerj.10997 (PMC8005288; doi:10.7717/peerj.10997)
Supplement: Supplemental Information 3 [file peerj-09-10997-s003.docx]

# Exploratory Analysis of Individual Behaviors in Experiment 1

We conducted a series of exploratory regressions using generalized estimating equations (GEE; Hardin & Hilbe, 2003) to discover the factors that were related to the probability of DLR, the average number of legs lifted during DLR, the probability of bite, and the probability of sting during each trial. For each regression, we initially included all other behaviors as factors in the model, as well as both groups, trial. We also included head size as a physical measure consistent with other literature (e.g. Riveros & Gronenberg, 2009b). Regressions were conducted using generalized estimating equations with a binomial link when analyzing probability of DLR, bite or sting, and a Gaussian link when analyzing number of legs lifted during DLR. We did not include interactions in our models. For all regressions, we considered the categorical group variable as two mutually exclusive variables rather than include one group in the intercept. This interceptless form of displaying the regression leads to a more symmetric interpretation of categorical parameters. For all variables, the parameter estimates and corresponding *p* values represent the difference from 50% for logistic regressions, or difference from zero for linear regressions. The group parameters in our interceptless regressions can also be directly compared by creating a z score by dividing the difference between the estimates by the square root of the sum of the squared standard errors of the estimates (Clogg, Petkova, & Haritou, 1995; Paternoster, Brame, Mazerolle, & Piquero, 1998). Parameters other than group were removed in a backwards elimination manner based on non-significant *p* values (≥ 0.05).

Table S1 shows the regression model for probability of DLR (top). We eliminated head size and trial, in that order, due to *p* values in the initial models of 0.755 and 0.541, respectively. The refined model shows that bees in the experimental group emitted significantly more DLR than chance, and that bees in the control group emitted almost significantly less DLR than chance (*p* = 0.05). A direct comparison of group parameters revealed that DLR occurred significantly more in the experimental group than the control group (estimate difference = 2.426, z = 4.456, *p* < 0.000). The analysis also revealed that DLR was significantly less likely than chance when a bite occurred, and significantly more likely when a sting occurred. Table S1 also shows the average number of legs lifted during DLR (bottom). We eliminated head size, trial, and bite in that order, due to *p* values in the initial models ranging from 0.965 to 0.222. The refined model shows that bees in both groups lifted significantly more than zero legs during trials, and a direct comparison of group parameters revealed that number of legs lifted was significantly higher in the experimental group than the control group (estimate difference = 0.914, z = 5.310, *p* < 0.000). The number of legs lifted also increased significantly in trials with a sting.

Table S1

| *DLR Probability* | | | | | |
| --- | --- | --- | --- | --- | --- |
| Parameter | Estimate | Standard Error | 95% Confidence Intervals | | *p* value |
| Experimental | 1.888 | 0.470 | 0.967 | 2.810 | 0.000 |
| Control | -0.538 | 0.275 | -1.077 | 0.001 | 0.050 |
| Bite | -1.391 | 0.442 | -2.257 | -0.524 | 0.002 |
| Sting | 2.276 | 0.809 | 0.690 | 3.862 | 0.005 |
| *DLR Average Legs Lifted* | |  |  |  |  |
| Parameter | Estimate | Standard Error | 95% Confidence Intervals | | *p* value |
| Experimental | 1.318 | 0.154 | 1.016 | 1.619 | 0.000 |
| Control | 0.404 | 0.077 | 0.253 | 0.555 | 0.000 |
| Sting | 0.696 | 0.174 | 0.355 | 1.037 | 0.000 |

The top portion of Table S2 shows the regression model for probability of biting, first including DLR as a nominal variable. We eliminated head size and trial, in that order, due to *p* values in the initial models of 0.860 and 0.833, respectively. The refined model shows that bees in the experimental group bit at around 50% of trials, bees in the control group bit significantly less than chance, and a direct comparison of group parameters revealed that biting occurred significantly more in the experimental group than the control group (estimate difference = 1.108, z = 2.058, *p* = 0.040). Biting was also significantly less likely than chance during trials with a DLR, but significantly more likely in trials with a sting. The bottom portion of Table S2 shows the regression where the nominal DLR factor was switched to the continuous number of legs lifted factor. The analysis shows that while there was a significant inverse relationship between DLR and bite, the number of legs lifted during DLR did not significantly impact bite probability. Instead, DLR and bite appear to be alternative behaviors.

Table S2

| *Bite Probability - Nominal DLR* | | | | | |
| --- | --- | --- | --- | --- | --- |
| Parameter | Estimate | Standard Error | 95% Confidence Intervals | | *p* value |
| Experimental | -0.154 | 0.441 | -1.019 | 0.711 | 0.727 |
| Control | -1.262 | 0.308 | -1.865 | -0.658 | 0.000 |
| DLR | -1.378 | 0.435 | -2.230 | -0.526 | 0.002 |
| Sting | 1.729 | 0.362 | 1.020 | 2.438 | 0.000 |
| *Bite Probability - DLR Legs Lifted* | | | | | |
| Parameter | Estimate | Standard Error | 95% Confidence Intervals | | *p* value |
| Experimental | -0.851 | 0.433 | -1.700 | -0.002 | 0.049 |
| Control | -1.493 | 0.319 | -2.119 | -0.868 | 0.000 |
| Legs Lifted | -0.238 | 0.217 | -0.664 | 0.188 | 0.274 |
| Sting | 1.566 | 0.392 | 0.798 | 2.334 | 0.000 |

Finally, Table S3 shows the regression model for probability of stinging, first including DLR as a nominal variable (top). We again eliminated trial and head size, in that order, due to *p* values in the initial models of 0.634 and 0.069, respectively. The refined model shows that bees in both the experimental and control groups stung significantly less than chance, and a direct comparison of group parameters revealed that stinging occurred significantly more in the experimental group than the control group (estimate difference = 4.429, z = ﻿3.398, *p* = 0.001). Stinging was also significantly more likely than chance on trials with a DLR as well as on trials with a bite. The bottom portion of Table S3 shows a similar regression where the nominal DLR factor was switched to the continuous number of legs lifted factor. The results of the two analysis are extremely similar indicating that the probability of stinging increases both when a DLR occurs, and as the number of legs lifted increases.

Table S3

| *Sting Probability - Nominal DLR* | | | | | |
| --- | --- | --- | --- | --- | --- |
| Parameter | Estimate | Standard Error | 95% Confidence Intervals | | *p* value |
| Experimental | -1.747 | 0.824 | -3.363 | -0.131 | 0.034 |
| Control | -6.176 | 1.009 | -8.154 | -4.197 | 0.000 |
| DLR | 2.312 | 0.841 | 0.664 | 3.960 | 0.006 |
| Bite | 1.942 | 0.414 | 1.131 | 2.753 | 0.000 |
| *Sting Nominal - DLR Legs Lifted* | | | | | |
| Parameter | Estimate | Standard Error | 95% Confidence Intervals | | *p* value |
| Experimental | -1.675 | 0.656 | -2.960 | -0.389 | 0.011 |
| Control | -5.592 | 0.748 | -7.058 | -4.125 | 0.000 |
| Legs Lifted | 1.240 | 0.364 | 0.526 | 1.954 | 0.001 |
| Bite | 1.802 | 0.453 | 0.914 | 2.690 | 0.000 |

In the regression model of probability of stinging including DLR as a nominal factor (Table S3, top) head size had the smallest *p* value of any factor removed from the models. Given documented effects of head size on behavior (e.g., Riveros & Gronenberg, 2009b), we also considered how weight (a closely correlated measure) and a linear density value as weight / head size (g / mm) would impact probability of stinging. While including head size in the model had a small positive effect (estimate = 0.8095, *p* = 0.069) on probability of sting, including weight in the model had a small, insignificant negative effect (estimate = -5.3309, *p* = 0.197). Interestingly, density had a very small, but significant effect on probability of sting (estimate = -28.2914, *p* = 0.013), however this inclusion removed the significant effect of the experimental group. Though it appears that, less dense bees (lower weight to head size ratio) were significantly more likely to sting, we suggest that this particular finding be interpreted with caution given the very small effect and highly exploratory nature of the analysis. It is perhaps best interpreted, not as a specific result for this experiment, but as suggestion that the continual measurement of physical proportions in bumble bee behavioral research may prove useful.

Taken together, the results of the exploratory analysis in Tables S1, S2 and S3 recapitulate the findings observed in Figures 2 and 3. Bees in the experimental group responded more, and trial had no impact on behavior. The regressions also suggest that DLR and sting are likely to occur together, but DLR and bite appear to be inversely associated.

# Analysis of Final Trials in Experiment 2

Table S4

| *Analysis of DLR by Trial* | | | | | |
| --- | --- | --- | --- | --- | --- |
| Parameter | Estimate | Standard Error | 95% Confidence Intervals | | *p* value |
| Captive Trial 1 | 0.379 | 0.360 | -0.326 | 1.085 | 0.292 |
| Captive Trial 10 | -1.946 | 0.535 | -2.994 | -0.898 | 0.000 |
| Captive Trial 11 | 0.000 | 0.354 | -0.693 | 0.693 | 1.000 |
| Captive Trial 12 | -2.269 | 0.606 | -3.457 | -1.080 | 0.000 |
| Captive Trial 13 | -0.125 | 0.354 | -0.819 | 0.569 | 0.724 |
| Wild Trial 1 | -0.511 | 0.365 | -1.227 | 0.205 | 0.162 |
| Wild Trial 10 | -2.269 | 0.606 | -3.457 | -1.080 | 0.000 |
| Wild Trial 11 | -0.379 | 0.360 | -1.085 | 0.326 | 0.292 |
| Wild Trial 12 | -1.686 | 0.487 | -2.641 | -0.732 | 0.001 |
| Wild Trial 13 | -0.379 | 0.360 | -1.085 | 0.326 | 0.292 |

# The Lost Bee Effect


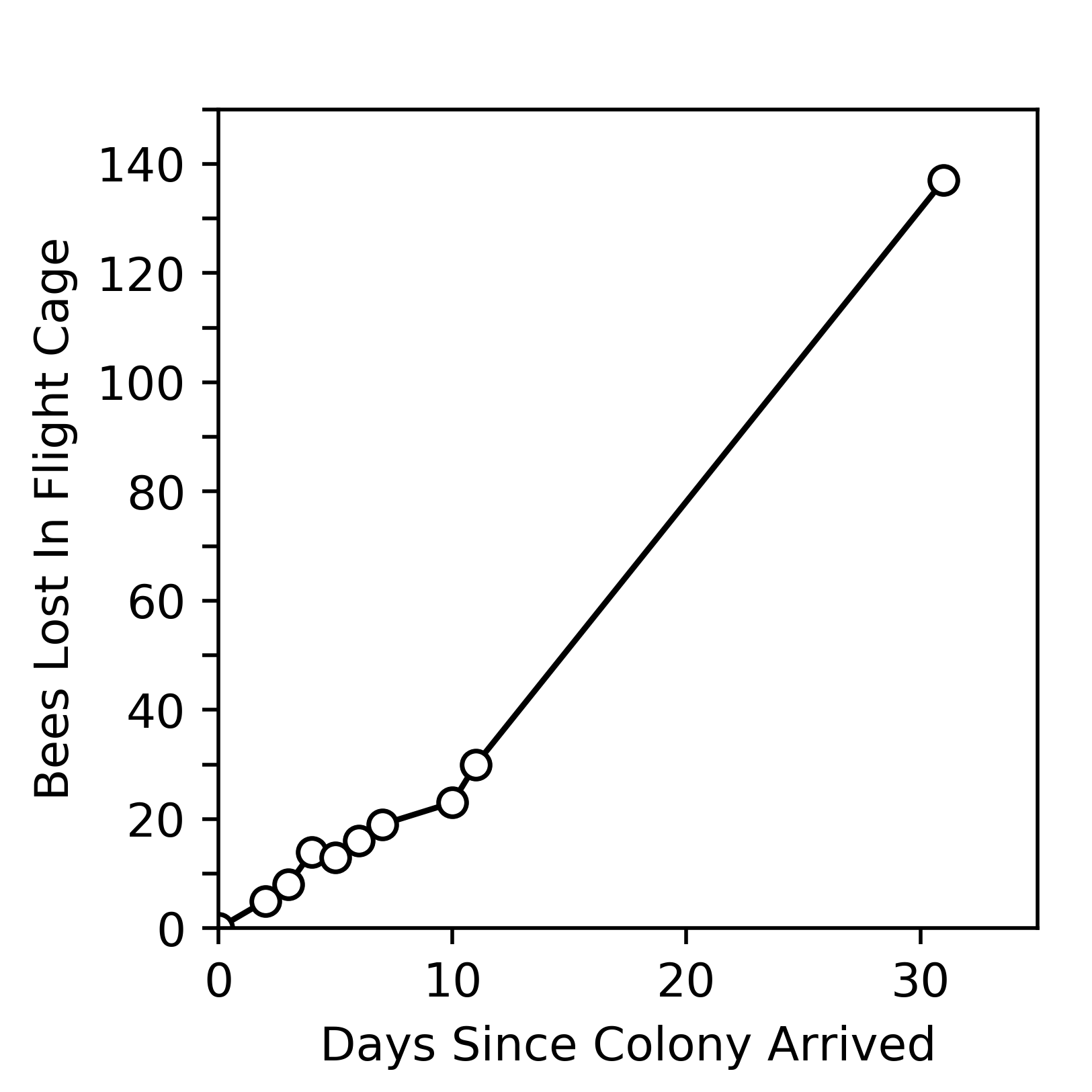


**Figure S1.** The lost bee effect. This data reports the number of bees that became isolated from their hive. The data shown here is from a colony with connected flight cage set up as reported in experiment 2. After a period of 31 days, over half the population of worker bees became isolated from the hive, while the queen and a small number of workers remained in the hive. Initially, bees were counted in the morning or evening when the laboratory lights and heat were off and the bees were inactive. After several weeks, the number of lost bees made this impractical. A final count was taken after research with this colony was complete. The entire flight cage was moved to the refrigerator so that the bees could be chilled, counted, weighed, measured, then returned to the colony. Similar patterns of lost bees were observed with other colonies maintained in the same manner.

# References

Clogg, C. C., Petkova, E., & Haritou, A. (1995). Statistical methods for comparing regression coefficients between models. *American Journal of Sociology, 100*(5), 1261-1293. doi: 10.1086/230638

Hardin J., Hilbe J.M. (2003). *Generalized Estimating Equations*. Boca Raton, FL: Chapman & Hall.

Paternoster, R., Brame, R., Mazerolle, P., & Piquero, A. (1998). Using the correct statistical test for the equality of regression coefficients. *Criminology*, *36*(4), 859 - 866.

Riveros, A. J., & Gronenberg, W. (2009b). Olfactory learning and memory in the bumblebee *Bombus occidentalis*. *Naturwissenschaften, 96*: 851-856. doi: 10.1007/s00114-009-0532-y
